# The response of gene expression associated with lipid metabolism, fat deposition and fatty acid profile in the *longissimus dorsi* muscle of Gannan yaks to different energy levels of diets

**DOI:** 10.1371/journal.pone.0187604

**Published:** 2017-11-09

**Authors:** Chao Yang, Jianbin Liu, Xiaoyun Wu, Pengjia Bao, Ruijun Long, Xian Guo, Xuezhi Ding, Ping Yan

**Affiliations:** 1 Key Laboratory of Yak Breeding Engineering, Lanzhou Institute of Husbandry and Pharmaceutical Sciences, Chinese Academy of Agricultural Sciences, Lanzhou, P.R. China; 2 State Key Laboratory of Pastoral Agricultural Ecosystem, College of Pastoral Agriculture Science and Technology, Lanzhou University, Lanzhou, P.R. China; 3 International Centre for Tibetan Plateau Ecosystem Management, Lanzhou University, Lanzhou, P.R. China; 4 School of Life Sciences, Lanzhou University, Lanzhou, P.R. China; University of Illinois, UNITED STATES

## Abstract

The energy available from the diet, which affects fat deposition *in vivo*, is a major factor in the expression of genes regulating fat deposition in the *longissimus dorsi* muscle. Providing high-energy diets to yaks might increase intramuscular fat deposition and fatty acid concentrations under a traditional grazing system in cold seasons. A total of fifteen adult castrated male yaks with an initial body weight 274.3 ± 3.14 kg were analyzed for intramuscular adipose deposition and fatty acid composition. The animals were divided into three groups and fed low-energy (LE: 5.5 MJ/kg), medium-energy (ME: 6.2 MJ/kg) and high-energy (HE: 6.9 MJ/kg) diets, respectively. All animals were fed *ad libitum* twice daily at 08:00–09:00 am and 17:00–18:00 pm and with free access to water for 74 days, including a 14-d period to adapt to the diets and the environment. Intramuscular fat (IMF) content, fatty acid profile and mRNA levels of genes involved in fatty acid synthesis were determined. The energy levels of the diets significantly (*P<*0.05) affected the content of IMF, total SFA, total MUFA and total PUFA. C16:0, C18:0 and C18:1n9c account for a large proportion of total fatty acids. Relative expression of acetyl-CoA carboxylase (*ACACA*), fatty acid synthase (*FASN*), stearoyl-CoA desaturase (*SCD*), sterol regulatory element-binding protein-1c (*SREBP-1c*), peroxisome proliferator-activated receptor γ (*PPAR*γ) and fatty acid-binding protein 4 (*FABP4*) was greater in HE than in LE yaks (*P<*0.05). Moreover, ME yaks had higher (*P<*0.05) mRNA expression levels of *PPAR*γ, *ACACA*, *FASN*, *SCD* and *FABP4* than did the LE yaks. The results demonstrate that the higher energy level of the diets increased IMF deposition and fatty acid content as well as increased intramuscular lipogenic gene expression during the experimental period.

## Introduction

Yaks (*Bos grunniens*), one of the world’s most extraordinary herbivores, are a multifunctional principal livestock species unique to the Qinghai-Tibetan Plateau, where altitudes range from 3,000 to 5,000 m and which is characterized by hypoxia, low annual average temperatures, a short forage growing season (approximately 90–120 days) and a shortage of forage nutrients [1, 2]. There are approximately 15 million yaks on the Qinghai-Tibetan Plateau, accounting for more than 90% of total yak population in the world. These yaks provide many major resources (e.g., meat, milk, hair, hides and dung), and yak meat is the major economic resource for local herders [3, 4].

As quality life improves worldwide, people increasingly expect that the meat they consume should be healthy and high quality (low in fat; high in protein, vitamins and minerals). The intramuscular fat (IMF) and intramuscular fatty acid content play an important role in meat quality [5, 6]. However, yak meat has a low IMF content because intramuscular adipose deposition is difficult under the conditions of long-term malnutrition in a traditional grazing system in cold seasons [7]. Meat quality, including palatability, tenderness and juiciness, could be improved by increasing the IMF content [8], and increasing the IMF content and fatty acid profile may improve the quality of yak meat. Many studies have demonstrated that the IMF content and fatty acid levels are influenced by age [9], breed [10] and diets [11–15]. *In vivo*, lipogenesis, lipolysis and fatty acid transport lead to IMF deposition. A higher-energy content diet could contribute to lipogenesis [16]. The concentration of intramuscular fatty acids is mainly regulated by inducing and inhibiting genes encoding specific metabolic enzymes associated with lipid metabolism or transcription factors [17]. Sterol regulatory element-binding protein-1c (*SREBP-1c*) and peroxisome proliferator-activated receptor γ (*PPARγ*) are the most important genes involved in lipid metabolism in muscle tissue [18, 19]. Recently, effects of different dietary energy levels on IMF deposition and fatty-acid composition in beef cattle [20], double-muscled Belgian Blue bulls [21] and Angus × Chinese Xiangxi yellow cattle [22] have been reported, as being affected by diets with different protein levels on intramuscular fat deposition in yaks [23]. However, little information is available regarding the influence of different energy diets on IMF deposition and fatty acid profiles in yaks.

The effects of different dietary energy content on IMF deposition and fatty acid composition in yaks are unclear. Therefore, the objective of this study was to analyze the relationship between dietary energy and fat deposition and fatty-acid profile, as well as expression of the regulatory genes *PPARγ*, *SREBP-1c*, stearoyl-CoA desaturase (*SCD*), acetyl CoA carboxylase (*ACACA*), lipoprotein lipase (*LPL*), fatty acid-binding protein 4 (*FABP4*) and fatty acid synthase (*FASN*) in the muscle of adult yaks.

## Materials and methods

### Ethics statement

This feeding experiment was conducted between February and May 2016 at Hongtu Yak Breeding Cooperatives (located in Qinghai-Tibetan Plateau, at 35°08′38″N, 102°99′36″E and with average altitude 3,230 m) of Tibetan Autonomous Prefecture of Gannan, Gansu Province, China. Before the experiment, all animal studies and the barn environment were examined and approved by the Institutional Animal Care and Use Committee of Lanzhou Institute of Husbandry and Pharmaceutical Sciences, China. The yaks were provided for use as experimental animals by the owner, and all animals were cared for according to the Guide for the Care and Use of Laboratory Animals, Lanzhou Institute of Husbandry and Pharmaceutical Sciences, China.

### Animals, diets and management

A total of fifteen adult castrated male yaks with similar body conditions (body weight 274.3 ± 3.14 kg) were included in a single-factor completely randomized experimental design. The animals were randomly divided into three treatment groups with five replicates each. Three diets with different levels of energy and a concentrate-to-forage ratio of 30:70 (DM basis) were formulated to be isonitrogenous, containing similar roughage mixtures (40% oats silage, 40% microbial corn stalk silage and 20% highland barley hay) and different energy concentrates: low energy level (LE: 5.5 MJ/kg), medium energy level (ME: 6.2 MJ/kg) and high energy level (HE: 6.9 MJ/kg). The ingredients and nutrient composition of the three diets are shown in Table 1.

**Table 1:** Ingredients and nutrient composition of the diets during the experiment.

Item	LE	ME	HE
Ingredient (%)			
Corn	29.00	44.80	56.00
Corn germ	30.00	20.00	12.00
Wheat bran	4.00	4.00	─
DDGS	15.00	7.00	6.30
Prickly ash seed	10.00	2.00	4.00
Cottonseed meal	6.00	12.00	16.00
Soybean meal	─	5.00	─
Salt	0.80	0.80	0.80
White stone powder	2.00	2.00	2.00
Dicalcium phosphate	0.60	0.60	0.60
Urea	0.80	─	0.50
Sodium bicarbonate	1.00	1.00	1.00
Premix^a^	0.80	0.80	0.80
Nutrient composition, % of DM			
Crude protein	16.53	16.74	17.21
Crude fat	3.73	4.18	5.57
NEg^b^ (MJ/kg)	5.5	6.2	6.9
Neutral detergent fiber	15.93	13.15	12.32
Acid detergent fiber	4.54	4.14	3.72
Calcium	0.64	0.84	0.75
Phosphorus	0.31	0.34	0.36

^a^ Premix was provided per kilogram of total diet DM, and the composition was as follows: 22,520 IU of vitamin A, 1,920 IU of vitamin D_3_, 18 IU of vitamin E, 0.36 IU of vitamin K_3_, 5.28 mg of vitamin B_2_, 0.008 mg of vitamin B_12_, 21.2 mg of D-calcium pantothenate, 9 mg of Cu, 132.8 mg of Zn, 240 mg of Fe and 8 mg of Mn, 0.28 mg of Co.

^b^ NEg, net energy for gain; DDGS, dried distillers grains with solubles; LE, low energy level; ME, medium energy level; HE, high energy level.

The experiment lasted for 60 d, and the animals were given a 14-d adaptation period to familiarize them with the diets, facilities and staff before the experiment. All animals were weighed, labeled before feeding in the morning and then individually housed in tie-stalls; each animal had 9 m^2^ for normal activities with bedding, and the barn was cleaned every day. The yaks were fed twice a day at 08:00–09:00 am and 17:00–18:00 pm with a total of 2.45 kg concentrates and 5.75 kg roughage mixtures; they were given free access to water and mineral blocks. The final weights of the animals were determined by weighing them after fasting them for 12 h. They were transferred to a slaughter house and all yaks received a jugular vein injection of sierra oxazine hydrochloride injection (Shengda Animal Pharmaceutical Co. Ltd., Dunhua, Jilin, China) with 0.2 mL/kg liveweight to ease their pain before slaughter, and exsanguination via the carotid artery was executed after animals completely under anesthesia.

### Sample collection

After slaughter, the carcasses were immediately washed and divided into halves. Subsequently, two samples of the *longissimus dorsi* muscle between 12th and 13th ribs were collected from each animal. All instruments used to collect the tissue were sterilized in advance. The muscle samples were washed in 0.9% NaCl solution, and the first samples were stored in Ziploc bags at -20°C for proximate nutrient and fatty acid analysis. The second were placed in 2 mL cryogenic vials (Corning Incorporated, New York, USA), transported in liquid nitrogen and stored at -80°C for total RNA extraction.

### Intramuscular fat content analysis

A 5-g meat sample was used to measure intramuscular fat content in each animal. Sea sand was added to evaporate moisture in a water bath. Lipids were extracted using the petroleum ether of Soxhlet apparatus (AOAC method) and expressed as grams per 100 g of fresh muscle tissue.

### Fatty acid profile analysis

Samples of yak muscles (100 mg) were trimmed of connective tissue and finely chopped. Intramuscular fat was extracted in 3 mL chloroform-methanol 1:1 [24] and methylated in 2 mL 4% methanol solution in HCl [25] containing 100 μL C19:0 (methyl nonadecanoate) as an internal standard, and then the mixtures were heated in a water bath at 85°C for 1 h. Subsequently, 1 mL n-hexane was added to the mixture after it reached room temperature and shaking extraction was performed for 2 min. The mixture was allowed to stand for 1 h until layering occurred; following this, 100 μL supernatant was transferred to a new centrifuge tube and diluted with n-hexane to 1 mL. The extract was then filtered through filter membrane with an aperture of 0.45 μm. Fatty acid profiles were determined by gas chromatograph-mass spectrometer (ThermoFisher Trace 1310, Thermo Scientific, MA, USA) with a flame ionization detector and a 30-m-long capillary column 0.25 mm in internal diameter and 0.25 μm thick (TG-5MS, Thermo Scientific, MA, USA). Nitrogen was used as a carrier gas at a flow rate of 1.2 mL/min. The chromatographic conditions were as follows: the capillary column was incubated at 80°C for 1 min, and the temperature was increased by 10°C/min until it reached 200°C. Subsequently, the temperature was increased by 4°C/min until it reached 250°C and then increased at 2°C/min to 270°C. Following this, the temperature was held for three minutes at 270°C. During analysis, the temperature of injector was kept at 290°C and splitless injection was performed with a 1-min opening valve time. Next, the capillary column was directly subjected to mass spectrum analysis under the following conditions: the interface temperature set at 290°C and the ion source maintained at 280°C. Otherwise, 70 eV ionization energy of impact ionization (EI) was used to fragment the eluents from the capillary gas chromatograph. The scanned area was from 30 to 400 amu (atomic mass units). Individual fatty acid contents were determined through comparison to the mixed fatty acid standard product and the internal standard, and the fatty-acid levels were calculated by following formula:

$$X_{i}=m*A_{\mathrm{si}}/A\sum si$$

where X_i_ is the level of each fatty acid, expressed as mg/kg muscle tissue; m is the intramuscular fat content of the sample; A_si_ represents the peak area of all types of fatty acids in the sample; *A* ∑ *si* is the sum of the peak area of all fatty acids in the sample.

### RNA extraction and quantitative RT-PCR

The target and reference gene primers were designed using integrated mRNA sequences based on sequences published by the National Center for Biotechnology Information (NCBI) (www.ncbi.nlm.nih.gov) primers were designed using Premier Primer 5 software (PREMIER Biosoft International, CA, USA), and sequences of each are shown in Table 2.

**Table 2:** Gene names, primer sequences, accession, product size of the used genes.

Gene name*	Primer sequence (5’→3’)		Accession	Product size
β-actin	F	ACCATCGGCAATGAGCG	XM_005887322.2	150bp
	R	CACCGTGTTGGCGTAGAG		
LPL	F	TCCTGGAGTGACCGAATC	XM_005902304.2	125bp
	R	AGGCAGCCACGAGTTTT		
ACACA	F	AAGCAATGGATGAACCTTCTTC	XM_005888165.2	197bp
	R	GATGCCCAAGTCAGAGAGC		
PPARγ	F	CATTTCCACTCCGCACTA	XM_005902845.2	122bp
	R	GGGATACAGGCTCCACTT		
FASN	F	GACGGTCGCATCATCTTCC	XM_005905364.2	156bp
	R	GAGCACAATCCCTGTCTTCG		
FABP4	F	TGAGATTTCCTTCAAATTGGG	XM_014478668.1	101bp
	R	CTTGTACCAGAGCACCTTCATC		
SCD	F	TACTGCGGTCCAAGTCGTT	NM_173959.4	165bp
	R	CAGCCTTGTCTGGAGTCATC		
SREBP-1c	F	AGCTCAAGGACCTGGTGGTG	XM_014477492.1	140bp
	R	GACAGCAGTGCGCAGACTCA		

* LPL, lipoprotein lipase; ACACA, acetyl-CoA carboxylase; PPARγ, peroxisome proliferator-activated receptors gamma; FASN, fatty acid synthase; FABP4, adipocyte fatty acid binding protein 4; SCD, stearoyl-CoA desaturase; SREBP-1c, sterol regulatory element-binding protein-1c.

The total RNA of the *longissimus dorsi* muscle was extracted by RNAiso Plus (TaKaRa, Dalian, China) according to the manufacturer’s instructions. The extracted RNA was dissolved in 30 μL diethylpyrocarbonate (DEPC)-treated water (Solarbio LIFE SCIENCES, Beijing, China), and the concentration was measured by spectrophotometer (NanoDrop 2000, Thermo Scientific, MA, USA). Purity and integrity were checked using agarose-gel electrophoresis with ethidium bromide. Total RNA was reverse transcribed into five copies using a PrimeScript™ RT reagent kit (TaKaRa, Dalian, China) in accordance with the manufacturer’s instructions, and the RT-PCR products were stored at -20°C for quantitative real-time PCR.

Quantitative real-time PCR was performed in triplicate to determine mRNA relative expression using a SYBR® Premix Ex Taq™ II (TaRaKa, Dalian, China). Each 25-μL real-time reaction contained 12.5 μL SYBR Premix Ex Taq II (2×), 1 μL each of 10 μM primers, 2.0 μL cDNA and 8.5 μL RNase Free dH_2_O. Reactions were run on a fluorescence thermal cycler (CFX96, Bio-Rad, CA, USA), and the program was as follows: 95°C for 30 s, 40 cycles of 95°C for 5 s, annealing at 60°C for 30 s and a melting curve with a temperature increase of 0.5°C every 5 s starting at 65°C. The RT-PCR analyses for each studied gene were performed using cDNA from five biological replicates with three technical replicates per biological replicate. The threshold cycle (C_T_) resulting from quantitative RT-PCR was analyzed using the 2^-ΔΔCt^ method, and all data were normalized with the reference gene beta-actin (*β*-actin) gene [26].

### Statistical analysis

The fatty acid content and mRNA abundance in the *longissimus dorsi* muscle were processed by one-way ANOVA analysis using the LSD procedure to perform multiple comparisons in SPSS 19.0 (SPSS Inc., Chicago, USA). The Pearson correlation analyses were performed to calculate correlation coefficients among gene expression, as well as correlation coefficients between gene expression and intramuscular fat content. Furthermore, the correlations between gene expression and fatty acid concentrations were also determined. Values at *P<*0.05 were considered significant difference.

## Results

### Intramuscular fatty acid composition

The effect of diets with different energy contents on the fatty acid profile of yaks is shown in Table 3. Intramuscular fat content was highly affected (*P* = 0.001) by dietary energy levels. The fatty acids content varied significantly among diets and was positively associated with higher-energy diets. Significant (*P<*0.001, *P* = 0.011 and *P* = 0.001) differences were found between HE and LE yaks regarding saturated fatty acids (SFA), monounsaturated fatty acids (MUFA) and polyunsaturated fatty acids (PUFA), and there were also significant (*P* = 0.037 and *P* = 0.005) differences between HE and ME yaks in terms of SFA and PUFA content. However, ME yaks were not significantly different from LE and HE yaks (*P* = 0.227 and *P* = 0.111) in terms of MUFA, and the level of PUFA was similar (*P* = 0.363) between the ME and LE yaks.

**Table 3:** IMF content and fatty acid composition in *longissimus dorsi* of yaks fed diets supplying different energy levels.

Item*	LE	ME	HE	SEM	*P*-value
IMF (g/100 g)	0.56^c^	0.92^b^	1.34^a^	0.102	0.001
Fatty acid (mg/kg)					
C14:0 Myristic acid	15.35^c^	21.55^b^	25.11^a^	1.138	*<*0.001
C14:1 Myristoleic acid	3.33^c^	4.14^b^	5.73^a^	0.291	*<*0.001
C15:0 Pentadecanoic acid	2.08^b^	3.01^a^^b^	4.03^a^	0.308	0.020
C16:0 Palmitic acid	1951.06^c^	2748.31^b^	3367.25^a^	174.237	*<*0.001
C16:1 Palmitoleic acid	69.80^c^	81.91^b^	118.14^a^	5.60	*<*0.001
C17:0 Margaric acid	6.31^b^	7.81^b^	10.26^a^	0.595	0.010
C17:1 Heptadecanoic acid	5.20^b^	6.02^a^^b^	8.44^a^	0.566	0.038
C18:0 Stearic acid	1044.74^c^	1242.88^b^	1588.32^a^	63.775	*<*0.001
C18:1n9c Oleic acid	1952.68^b^	2441.40^a^^b^	3059.18^a^	186.659	0.038
C18:1n9t *trans* oleic acid	37.37^b^	44.45^b^	75.05^a^	5.866	0.008
C18:2n6c Linolenic acid	64.49^b^	66.39^b^	80.41^a^	2.846	0.029
C20:1 Eicosenoic acid	1.22^b^	1.82^a^	2.11^a^	0.136	0.012
C20:3n3 Eicosatrienoic acid	0.71^b^	1.14^a^	1.31^a^	0.085	0.002
C20:4n6 Arachidonic acid	8.61^c^	11.81^b^	18.81^a^	1.185	*<*0.001
C20:5n3 EPA	1.84^b^	2.22^a^^b^	3.08^a^	0.212	0.036
C22:6n3 DHA	1.26^b^	1.54^a^^b^	2.17^a^	0.157	0.038
∑ SFA	3019.55^c^	4023.57^b^	4994.97^a^	232.906	*<*0.001
∑ MUFA	2069.60^b^	2579.74^a^^b^	3268.65^a^	195.509	0.027
∑ PUFA	76.91^b^	83.09^b^	105.78^a^	4.134	0.002

^a,b,c^ Means in a row with different small letter superscripts differ significantly (*P<*0.05).

* IMF, intramuscular fat; ∑ SFA, saturated fatty acid (without any double bonds, C14:0-C18:0); ∑ MUFA, monounsaturated fatty acid, all fatty acids with a single double bond (C14:1-C20:1); ∑ PUFA, polyunsaturated fatty acid, all fatty acids with 2 or more double bonds (including C18:2n6c, C20:3n3, C20:4n6 and C20:5n3); EPA, eicosapentaenoic acid; DHA, docosahexaenoic acid.

LE, low energy level; ME, medium energy level; HE, high energy level.

The fatty acids C16:0, C18:0 and C18:1n9c stand out, accounting for a large proportion of total fatty acids. HE yaks had the highest levels of C14:0, C14:1, C16:0, C16:1, C17:0, C18:0, C18:1n9t, C18:2n6c and C20:4n6, and HE yaks had significantly higher (*P<*0.001) concentrations of C14:0, C14:1, C15:0, C16:0, C16:1, 17:0, C17:1, C18:0, C18:1n9c, C18:1n9t, C18:2n6c, C20:1, C20:3n3, C20:4n6, C20:5n3 and C22:6n3 than did the LE yaks. Otherwise, significant (*P<*0.001, *P* = 0.022, *P* = 0.003, *P* = 0.001, *P* = 0.005, *P* = 0.034, *P* = 0.008 and *P* = 0.003) differences were found between ME and LE yaks in the concentrations of C14:0, C14:1, C16:0, C16:1, C18:0, C20:1, C20:3n3 and C20:4n6.

### mRNA abundance of *longissimus dorsi* muscle

**Fig 1:**
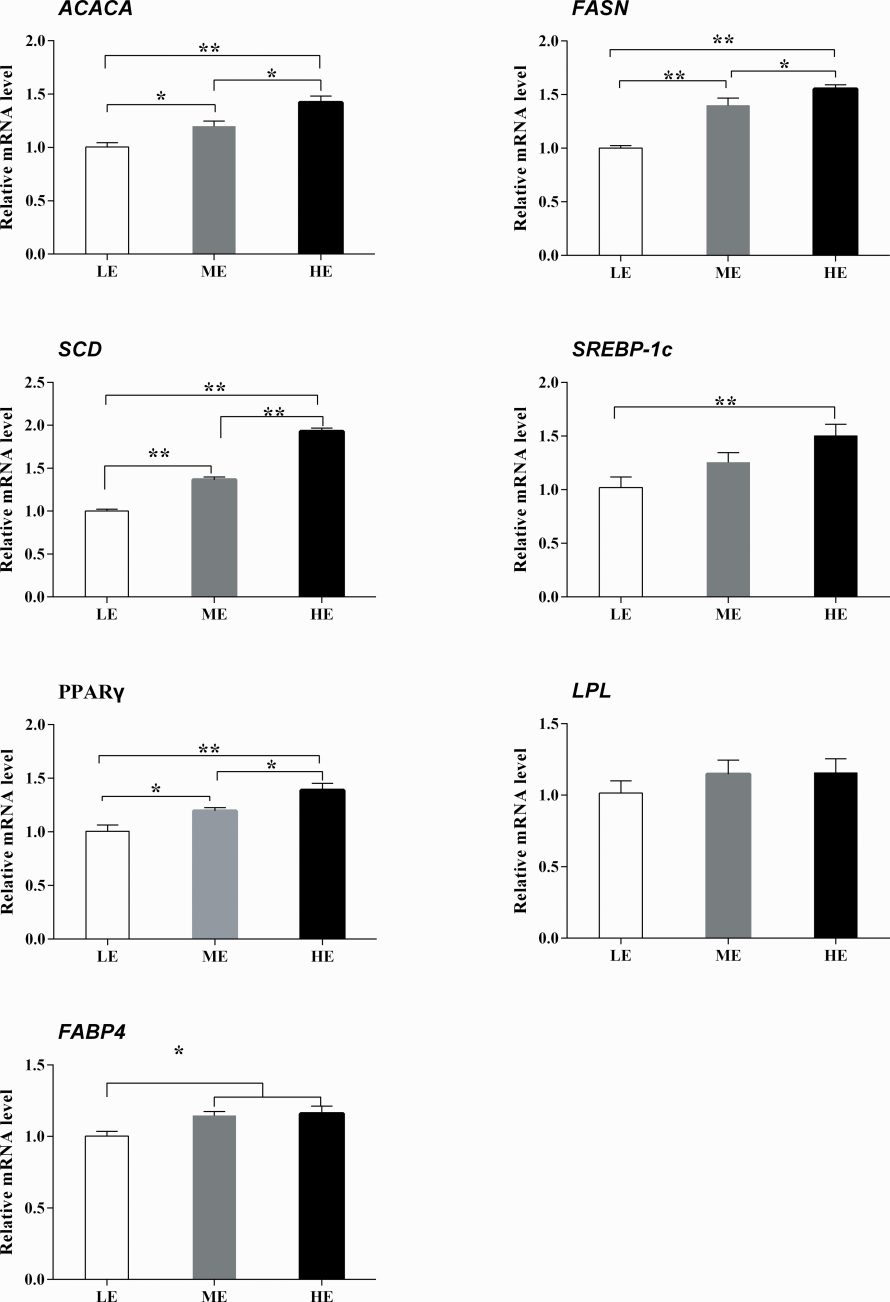
Relative expression levels of seven candidate genes in *longissimus dorsi* muscle of yaks fed diets supplying different energy levels. ACACA: acetyl-CoA carboxylase, FASN: fatty acid synthase, SCD: stearoyl-CoA desaturase, SREBP-1c: sterol regulatory element-binding protein-1c, PPARγ: peroxisome proliferator-activated receptors gamma, LPL: lipoprotein lipase, FABP4: fatty acid binding protein 4. Values are represented as the Mean ± SEM. * and ** indicate *P <* 0.05 and *P <* 0.01, respectively, between two groups.

The mRNA abundance of the candidate genes in each diet is shown in Fig 1. The mRNA levels of *ACACA*, *FASN*, *SCD*, *SREBP-1c*, *PPAR*γ and *FABP4* increased (*P<*0.05) with increasing dietary energy; HE and ME yaks had higher (*P<*0.05) mRNA abundance of above genes (except for *SREBP-1c*) than did the LE yaks. In addition, significant (*P* = 0.025, *P* = 0.01, *P* = 0.041 and *P<*0.001) differences were found in gene expression between HE and ME yaks for *PPAR*γ, *ACACA*, *FASN* and *SCD*; ME yaks had higher (*P<*0.001, *P<*0.001, *P<*0.001, *P<*0.001 and *P* = 0.016) expression levels of *PPAR*γ, *ACACA*, *FASN*, *SCD* and *FABP4* than did the LE yaks. Furthermore, no significant (*P* = 0.508) differences were found of *LPL* among three treatments, and HE and ME yaks had similar (*P>*0.05) expression of *SREBP-1c* and *FABP4*.

### Relationships among the expression levels of genes involved in lipid metabolism in *longissimus dorsi* muscle

The expression of *PPAR*γ was positively correlated with those of *ACACA* (*P<*0.01), *FABP4* (*P<*0.01), *SCD* (*P<*0.01), *SREBP-1c* (*P<*0.05) and *FASN* (*P<*0.01). The expression of *SREBP-1c* was also positively correlated with those of *ACACA* (*P<*0.01), *SCD* (*P<*0.05) and *FASN* (*P<*0.01). In addition, *FASN* expression was positively correlated with those of *ACACA* (*P<*0.01), *FABP4* (*P<*0.05) and *SCD* (*P<*0.01). There was a significant (*P<*0.01) correlation between *SCD* and *ACACA* and a positive correlation (*P<*0.05) between *SCD* and *FABP4* (Table 4).

**Table 4:** Pearson correlation coefficients among gene expression in *longissimus dorsi* muscle of yaks.

Gene ^a^	ACACA	LPL	FABP4	SCD	SREBP-1c	FASN
PPARγ	0.682**	0.075	0.730**	0.812**	0.534*	0.663**
FASN	0.684**	0.246	0.571*	0.850**	0.765**	
SREBP-1c	0.741**	0.220	0.267	0.631*		
SCD	0.748**	0.286	0.621*			
FABP4	0.351	-0.213				
LPL	0.407					

Pearson correlation coefficients are across all treatments. Number of observations = 15.

*P<0.05

** *P<*0.01,and

*** *P<*0.001.

^a^ LPL, lipoprotein lipase; ACACA, acetyl-CoA carboxylase; PPARγ, peroxisome proliferator-activated receptors gamma; FASN, fatty acid synthase; FABP4, adipocyte fatty acid binding protein 4; SCD, stearoyl-CoA desaturase; SREBP-1c, sterol regulatory element-binding protein-1c.

### Relationships among gene expression levels, intramuscular fat content and fatty acid content in *longissimus dorsi* muscle

IMF content was positively correlated with the expression levels of *PPAR*γ (*P<*0.05), *ACACA* (*P<*0.01), *FASN* (*P<*0.01), *SCD* (*P<*0.01) and *SREBP-1c* (*P<*0.01), but no significant correlation was found between IMF content and the expression of other genes (Table 5).

**Table 5:** Pearson correlation coefficients among gene expression, IMF and fatty acid content in *longissimus dorsi* muscle of yaks.

Item ^a^	PPARγ	FASN	ACACA	LPL	SREBP-1c	SCD	FABP4
IMF (g/100 g)	0.549*	0.791**	0.743**	0.339	0.785**	0.786**	0.216
Fatty acid ^b^ (mg/kg)							
C14:0 Myristic acid	0.735**	0.959**	0.753**	0.356	0.736**	0.887**	0.566*
C14:1 Myristoleic acid	0.693**	0.819**	0.788**	0.212	0.743**	0.889**	0.422
C16:0 Palmitic acid	0.805**	0.911**	0.710**	0.129	0.668**	0.851**	0.588*
C16:1 Palmitoleic acid	0.724**	0.812**	0.825**	0.244	0.730**	0.920**	0.481
C18:0 Stearic acid	0.762**	0.838**	0.830**	0.175	0.754**	0.886**	0.584*
C18:1n9c Oleic acid	0.446	0.896**	0.524*	0.177	0.224	0.651**	0.313
C18:1n9t *trans* oleic acid	0.489	0.727**	0.600*	-0.055	0.500	0.666**	0.267
C18:2n6c Linolenic acid	0.586*	0.672**	0.313	0.036	0.400	0.670**	0.309
C20:1 Eicosenoic acid	0.434	0.869**	0.447	0.404	0.360	0.750**	0.373
C20:3n3 Eicosatrienoic acid	0.776**	0.780**	0.466	0.187	0.449	0.853**	0.673**
C20:4n6 Arachidonic acid	0.822**	0.778**	0.744**	0.282	0.589*	0.961**	0.581*
C20:5n3 EPA	0.624*	0.779**	0.382	0.337	0.396	0.727**	0.291
C22:6n3 DHA	0.629*	0.776**	0.379	0.358	0.378	0.723**	0.289
∑ SFA	0.696**	0.913**	0.641*	0.096	0.544*	0.785**	0.524*
∑ MUFA	0.443	0.891**	0.532*	0.165	0.248	0.652**	0.304
∑ PUFA	0.711**	0.757**	0.472	0.140	0.488	0.819**	0.419

Pearson correlation coefficients are across all treatments. Number of observations = 15.

*P<0.05

** *P<*0.01, and

*** *P<*0.001.

^a^ ACACA, acetyl-CoA carboxylase; FASN, fatty acid synthase; SCD, stearoyl-CoA desaturase; SREBP-1c, sterol regulatory element-binding protein-1c; PPARγ: peroxisome proliferator-activated receptors gamma; LPL, lipoprotein lipase; FABP4, adipocyte fatty acid binding protein 4.

^b^ IMF, intramuscular fat; ∑ SFA, total saturated fatty acid (without any double bonds, C14:0-C18:0); ∑ MUFA, total monounsaturated fatty acid, all fatty acids with a single double bond (C14:1-C20:1); ∑ PUFA, total polyunsaturated fatty acid, all fatty acids with 2 or more double bonds (including C18:2n6c, C20:3n3, C20:4n6 and C20:5n3); EPA, eicosapentaenoic acid; DHA, docosahexaenoic acid.

Considering the correlations between gene expression and fatty acid content, *FASN* and *SCD* are notable (Table 5). These genes were positively correlated with levels of C14:0 (*P<*0.01), C14:1 (*P<*0.01), C16:0 (*P<*0.01), C16:1 (*P<*0.01), C18:0 (*P<*0.01), C18:1n9c (*P<*0.01), C18:1n9t (*P<*0.01), C18:2n6c (*P<*0.05 and *P<*0.01), C20:1 (*P<*0.01), C20:3n3(*P<*0.01), C20:4n6 (*P<*0.01), C20:5n3 (*P<*0.01), C22:6n3 (*P<*0.01), SFA (*P<*0.01), MUFA (*P<*0.01) and PUFA (*P<*0.01). The expression levels of *ACACA* and *PPAR*γ were positively correlated with C14:0 (*P<*0.01), C14:1 (*P<*0.01), C16:0 (*P<*0.01), C16:1 (*P<*0.01), C18:0 (*P<*0.01), C20:4n6 (*P<*0.01) and SFA (*P<*0.05 and *P<*0.01), and ACACA was positively correlated with C18:1n9c (*P<*0.05), C18:1n9t (*P<*0.05) and MUFA (*P<*0.05). *PPAR*γ had positive correlation with C18:2n6c (*P<*0.05), C20:3n3 (*P<*0.01), C20:5n3 (*P<*0.05), C22:6n3 (*P<*0.05) and PUFA (*P<*0.01). In addition, *SREBP-1c* was positively correlated with C14:0 (*P<*0.01), C14:1 (*P<*0.01), C16:0 (*P<*0.01), C16:1 (*P<*0.01), C18:0 (*P<*0.01), C20:4n6 (*P<*0.05) and SFA (*P<*0.05). Significant correlations were found between the expression level of *FABP4* and the concentrations of C14:0, C16:0, C18:0, C20:4n6, SFA (*P<*0.05) and C20:3n3 (*P<*0.01).

## Discussion

This paper describes for the first time the effects of diets supplying different energy levels on fat deposition and the fatty acid profile of the *longissimus dorsi* muscle in the domesticated yak and the role of genes involved in lipid metabolism in changing the fatty acid composition across three treatments. Non-structural carbohydrate (starch) was priority to support skeletal and muscle growth resulting in low rate of fat deposition during the “growing phase” in beef cattle. Conversely, the intake of starch, first and foremost, contributed to fat deposition during the “finishing phase” of beef cattle [27]. Consistently, several studies have demonstrated that IMF content increases with increased energy content in finishing cattle [20] and buffalo cattle [28], which was also found in current study. Bovine intramuscular fat is characterized by its abundant saturated fatty acids (SFA), especially palmitic acid and stearic acid [29], as we found for the yaks. Moreover, previous research showed that the level of SFA depends on the degree of fat deposition [30, 31] and that SFA content increased with increasing IMF content. It is, therefore, possible that higher energy content regulates SFA levels by enhancing IMF content. Consistent with our data, Smet et al [21] reported that the levels of C14:0, C15:0, C16:0, C16:1 and C18:1 increased with higher-energy diets in Belgian Blue bulls. By contrast, the level of C18:0 decreased in previous studies [32–34] but increased in the current research when higher-energy diets were provided. Pentadecanoic acid (C15:0) and heptadecanoic acid (17:0) belong to odd-chain fatty acids (OCFAs) and only have a small proportion of total saturated fatty acids in ruminant meat. *De novo* synthesis of linear OCFAs is different from palmitic acid which used propionyl-CoA as primer, instead of acetyl-CoA and a large portion of OCFAs in ruminants was synthesized by rumen bacteria, and it also originates from diets including maize silage, grass silage and other plants [35, 36]. Therefore, the synthesis of OCFAs may either not occur in adipose tissue or regulate by lipogenic genes.

Fatty acid composition of the *longissimus dorsi* muscle is affected by diets, including protein content, energy content [37] and fatty acid profile [17, 38, 39]. Abundant MUFA and PUFA not only improve meat characteristics, such as flavor, cholesterol content and nutritional benefits, but are also beneficial for human health. Contrary to our expectations, dietary MUFA and PUFA can’t be directly deposited in muscle due to the biohydrogenation of the rumen, and a portion of unsaturated fatty acids (UFA) become saturated [40]. The concentrations of MUFA and PUFA were greater (*P<*0.05) in the HE group than in the LE group, which might be influenced by two factors: On one hand, the diets contained different amounts of dried distillers grains with solubles (DDGS), and a previous study found that high-fat DDGS is a good source of UFA and to some extent, protects the UFA from ruminal biohydrogenation [41]. On the other hand, high dietary energy level may affect the normal function of rumen microbes associated with biohydrogenation, allowing more UFA to reach the small intestine, where they can be absorbed by the intestinal epithelium and deposited in muscle [42]. There were small amounts of eicosapentaenoic acid (EPA) and docosahexaenoic acid (DHA) in yak muscle, as described by Qin et al.; however, EPA and DHA were not detected in finishing cattle [22, 43]. To some degree, yak meat is more nutritious than cattle, as EPA and DHA contribute to reduced serum levels of fat and cholesterol and improved brain function [44]. In the HE condition, the SFA content was higher than those of MUFA and PUFA, and the meat therefore does not meet human nutritional needs. Therefore, future research should focus on developing yak meat with higher MUFA and PUFA levels and lower SFA levels.

IMF deposition is the result of a balance between dietary energy content and animal’s maintenance requirements. The net energy for gain (NEg) provided in the three diets was 5.5 MJ/kg, 6.2 MJ/kg and 6.9 MJ/kg. As a result, the high-energy diet had the highest IMF content. It is possible that more available substrate (glucose or acetate) involved in fat synthesis resulted in a higher degree of intramuscular fat deposition [45]. Additionally, the fatty acids profile of bovines is not strongly affected by diet because most dietary unsaturated fatty acids are saturated by microbial biohydrogenation in the rumen [46, 47]. Our results differed because higher-energy diets were provided. Fatty acid composition may be regulated by genes involved in lipid synthesis and fatty acid metabolism. Nevertheless, no studies have evaluated in detail the molecular mechanism underlying changes in fatty acid composition in finishing yaks. We found that expression of lipogenic and fatty acid metabolic genes affect fatty acid concentrations. Therefore, more attention should be paid to transcription factors and other key proteins related to lipid metabolism in the *longissimus dorsi* muscle.

The process of IMF deposition can be regulated by diets (including different energy or protein levels) through the potentially complex regulation mechanism. *PPARγ*, which belongs to the nuclear receptor superfamily, plays a vital role in regulating the expression of some genes encoding proteins involved in fat accumulation and adipocyte differentiation in adipose and muscle tissue. It has been reported that *PPARγ* was identified as an important candidate gene which has positive role in the several lipogenesis-related pathways of intramuscular fat using protein-protein interaction networks [48]. In detail, *PPARγ* promotes the expression of some adipocyte proteins or enzymes, such as fatty acid binding protein (*FABP4*), fatty acid synthase (*FASN*) and lipoprotein lipase (*LPL*) [49]. Few studies examined the effects of dietary energy levels on *PPARγ* gene expression in *longissimus dorsi* muscle of yak. We observed that the mRNA expression levels of *PPARγ*, *FABP4* and *FASN* were increased with increasing IMF content in the HE diets as compared to LE diet, but no expression changes occurred for the *LPL* gene. The result was in line with a previous study where the expressions of *PPARγ*, *FABP4* and *FASN* were activated with an increase in IMF content in Angus×Simmental cattle fed high-starch or low-starch diets [50], and *in vitro* study also found that bovine perimuscular pre-adipocytes cultured with insulin and dexamethasone to stimulate the differentiation had greater mRNA expression of *PPARγ*, *FABP4* and *FASN* compared with control [51]. It was expected that the expression of *PPARγ* was positively (*P<*0.05) correlated with the expression of *FABP4* and *FASN* as well as IMF content, however, had no correlation with *LPL* in our study. Interestingly, the expression of *LPL* was not significantly (*P>*0.05) influenced by dietary energy content in this study, indicating that the rate of lipogenesis was greater than that of lipolysis due to adequate energy intake and that little triglyceride was hydrolyzed into non-esterified fatty acids in the muscle to provide energy for the peripheral tissues. In brief, our results indicated that the IMF deposition in yaks fed high dietary energy was attributed to increased expression of *PPARγ*, *FABP4* and *FASN* which accelerate the rate of lipogenesis and low expression level of *LPL* during the finishing stage.

Peroxisome proliferator-activated receptors (*PPARs*), which belong to the nuclear receptor superfamily, are responsible for nutrient metabolism and energy homeostasis [52, 53]. All isoforms of PPARs can be activated or inhibited by the agonists (long-chain fatty acids or chemically associated derivatives) binding in the area of nutrition stems, and C16:0, C18:0, C18:1, C18:2 and C20:5n3 can indirectly activate *PPARγ* [54]. Long-chain fatty acids (LCFAs) such as C18:1 and C18:2, provided ligand to activate *PPARγ* and increased its mRNA expression level, can stimulate porcine adipocyte differentiation [55]. The significant positive correlations were found between the mRNA expression of *PPARγ* and the concentrations of C16:0, C18:2, C20:3n3, C20:4n6, C20:5n3 and C22:6n3 in our study. We speculated that these LCFAs activate the *PPARγ* to up-regulate its downstream genes and expedite the process of lipogenesis in intramuscular fat. *FABP4* is a carrier protein binding intracellular LCFAs into nucleus to activate peroxisome proliferator-activated receptors. The mRNA expression level of *FABP4* positively correlated with C16:0, C20:3n3 and C20:4n6 may be due to its greater affinity for these LCFAs.

It is well known that *FASN* produces a multipurpose enzyme that catalyzes the entire pathway of palmitate synthesis from malonyl-CoA [56]. In agreement with our results, Saburi et al [57] observed that *FASN* affected the synthesis of C14:0, C16:0, C16:1, C18:0, C18:1 and C18:2 as well as the ratio of monounsaturated to saturated fatty acids in Japanese black cattle and its TW haplotype enhanced C18:0, C18:1 content and decreased C14:0, C14:1, C16:0 and C16:1 content. The ‘AA’ genotype of the *FASN* SNP was dramatically related to higher concentrations of C14:0, C14:1, C16:1 and C18:2, but lower concentrations of 18:1n9c and C20:3n6 in Canadian commercial cross-bred beef steers [58]. However, we found the mRNA expression of FASN was positively (*P<*0.05) correlated with the content of large proportion of fatty acids which detected in current study. It suggested that the mechanism of *FASN* to regulate fatty acid synthesis may be influenced by different diets and genotypes or by the regulation of transcription factors (*PPARγ* and *SREBP-1c*).

In IMF accumulation, *SREBP-1c* is a critical transcription factor that regulates the expression of lipogenic genes (*ACACA*, *FASN* and *SCD*), and positive (*P<*0.05) correlations were found among these genes in current study (Table 4). The expression of *FASN*, *ACACA*, *SREBP-1c* and *SCD* was positively (*P<*0.05) correlated with IMF content, in agreement with Jeong and Kwon [59], who observed that expression of *ACACA* and *FASN* were significantly positively correlated (*P<*0.05) with IMF content and that higher IMF content up-regulated the expression of *FASN*, *ACACA*, *SREBP-1c* and *SCD* [37]. By contrast, no clear correlation between these genes and the IMF content of beef cattle fed soybeans or rumen-protected fat [17] may be due to higher dietary energy up-regulating the expression of genes related to lipid metabolism to enhance the rate of fat synthesis in our study. Acetyl CoA carboxylase (*ACACA*), a crucial rate-limiting enzyme in the mammalian cytosol, catalyzes the first step in *de novo* fatty-acid synthesis in the short term [60], resulting in the biosynthesis of long-chain fatty acids, principally palmitic acid. In this study, C16:0, C18:1n9t and C20:4n6 were positively associated with *ACACA*, but *ACACA* was not positively associated with C16:0 or other long-chain fatty acids in beef cattle fed high- or low-silage diets [61]. Two closely synonymous mutations in the *ACACA* gene significantly affected the polyunsaturated/saturated fatty acid ratio in the meat [62]. Therefore, the effect of *ACACA* on fatty acid composition may have been not evident because of single-nucleotide polymorphisms. Stearoyl-CoA desaturase (*SCD*) is the rate-limiting enzyme and catalyzes biosynthesis of MUFA from SFA, as well as conjugated linoleic acid from vaccenic acid. *SCD* increased the concentrations of C16:1 and C18:1 via C16:0 and C18:0 as substrates [63], which is similar to our results. However, *SCD* was negatively correlated with α-linolenic and arachidonic acid (C20:4n6) concentrations in beef cattle, and feeding linolenic acid (C18:2n6c), eicosapentaenoic acid (C20:5n3), and docosahexaenoic acid (C22:6n3) reduced *SCD* expression in rats by 50% [17, 64]. The results of this study, in which the expression of *SCD* was positively correlated with levels of C18:2n6c, C20:4n6 and C20:1, indicate that gene expression and fatty acids content might be primarily affected by dietary energy. *SREBP-1c* affects fatty acid composition through binding the sterol-regulatory-element sequences in the *SCD* promoter region [65], but the positive correlation between the expression of *SREBP-1c* and C20:4n6 levels may also be attributed to the diet. This suggested that the increased IMF content by high dietary energy level probably is due to increased expression of *SREBP-1c*, *FASN*, *ACACA* and *SCD*, and the above genes may further regulate the biosynthesis of fatty acids.

## Conclusions

The present study investigated the impact of dietary energy on the IMF deposition, partial fatty acid content and the mRNA expression of lipogenic genes in the *longissimus dorsi* muscle during the experimental period in detail. The results indicated that the HE diets promoted the deposition and partial fatty acid content of *longissimus dorsi* muscle mainly by up-regulation of mRNA expression of *ACACA*, *SCD*, *FASN*, *SREBP-1c*, *PPARγ* and *FABP4*. The data might be used to manipulate or provide useful information to improve IMF deposition and fatty acids accumulation in muscle.

## Supporting information

S1 File Experimental animals use permission. (PDF)

S1 Table Sequences of PCR product for each gene. (DOCX)
