# Supplementary material for: The response of gene expression associated with lipid metabolism, fat deposition and fatty acid profile in the longissimus dorsi muscle of Gannan yaks to different energy levels of diets
Source: PLoS One. 2017 Nov 9;12(11):e0187604. doi: 10.1371/journal.pone.0187604 (PMC5679530; doi:10.1371/journal.pone.0187604)
Supplement: S1 File — (PDF) [file pone.0187604.s001.pdf]

Experimental Animals Use Permission

The study executed by Dr. Xuezhi Ding and Mr. Chao Yang was conducted between February and May 2016 at Hongtu Yak Breeding Cooperatives (located in Qinghai-Tibetan Plateau, at 35°08′38″N, 102°99′36″E and with average altitude 3230 m) of Tibetan Autonomous Prefecture of Gannan, Gansu Province, China. Fifteen adult castrated Gannan yaks were used in this study. All experimental methods, animal care and the barn environment of this study were in strict accordance with the Guide for the Care and Use of Laboratory Animals, Lanzhou Institute of Husbandry and Pharmaceutical Sciences, China. In addition, all slaughter experiment was performed under anesthesia, and all efforts were made to minimize suffering. Thus, we agree to carry out the experiment and the certificate number was SCXK (Gan) 2014-0002.

Lanzhou Institute of Husbandry and Pharmaceutical Sciences

Chinese Academy of Agricultural Sciences

January 20, 2016
