# Supplementary material for: The response of gene expression associated with lipid metabolism, fat deposition and fatty acid profile in the longissimus dorsi muscle of Gannan yaks to different energy levels of diets
Source: PLoS One. 2017 Nov 9;12(11):e0187604. doi: 10.1371/journal.pone.0187604 (PMC5679530; doi:10.1371/journal.pone.0187604)
Supplement: S1 Table — (DOCX) [file pone.0187604.s002.docx]

| Gene name^*^ | Sequences of PCR product | Accession | Product size |
| --- | --- | --- | --- |
| β-actin | CACCGTGTTGGCGTAGAGGTCCTTGCGGATGTCGACGTCACACTTCATGATGGAATTGAAGGTAGTTTCGTGAATGCCGCAGGATTCCATGCCCAGGAAGGAAGGCTGGAAGAGAGCCTCAGGGCAGCGGAACCGCTCATTGCCGATGGT | XM_005887322.2 | 150bp |
| LPL | TCCTGGAGTGACCGAATCTGTGGCTAACTGTCACTTCAACCACAGCAGCAAAACCTTTGTGGTGATCCATGGCTGGACGGTGACAGGAATGTATGAGAGTTGGGTGCCAAAACTCGTGGCTGCCT | XM_005902304.2 | 125bp |
| ACACA | GATGCCCAAGTCAGAGAGCGTGTCTGAGCTGACCGAGGCTGGCGACAGAGAGCCCTCCTTCTCCTCCAGCAGGTCGAGCTTCACCAGGTTGCTAATCTCATCCTCCGAGTTATCTTCAGACACGGAGCCAATAATGAATCGGGAGTGCTGGTTCAGCTCCAGAGGTTTGGCCAAGGAAGAAGGTTCATCCATTGCTT | XM_005888165.2 | 197bp |
| PPARγ | CATTTCCACTCCGCACTATGAGGACATTCCGTTCCCAAGAGCTGACCCGATGGTTGCAGATTATAAGTATGACCTGAAGCTCCAAGAGTACCAAAGTGCAATCAAAGTGGAGCCTGTATCCC | XM_005902845.2 | 122bp |
| FASN | GAGCACAATCCCTGTCTTCGGCAGGATGACAGCCTGGTGCAGCGTCACGTCCTCGAATACTACAGGCGTGTGCTCCATGTTCTGGTCCAGGGCTCGGGCCAGTGTCTTCCAGACCAGGCACAGGTAGCCAGTGCCCGGGAAGATGATGCGACCGT | XM_005897713.1 | 156bp |
| FABP4 | CTTGTACCAGAGCACCTTCATCTAAGTTTACGATGCTCTTGACTTTCCTGTCATCTGGAGTGACTTCATCAAATTCCTGGCCCAATTTGAAGGAAATCTCA | XM_014478668 | 101bp |
| SCD | TACTGCGGTCCAAGTCGTTCCCTCTCTTACCGACAGAATGGTCAGGGTCACTGAACCACTGTTTCTCTTTACAAAGTTGAGCAAGCTGCCACTTTCACTTGGCCTCCAGAGTCTCCGTCTATATCCTTGTGCTCCTTACCACACTGATGACTCCAGACAAGGCTG | NM­_173959.4 | 165bp |
| SREBP-1c | GACAGCAGTGCGCAGACTCAGGTTCTCCTGCTTGAGCTTCTGGTTGCTGTGCTGAAGGAAGCGGATGTAGTCGATGGCCTTGCGCAAGACGGCAGATTTATTCAACTTGGCCTCGGTGCCCACCACCAGGTCCTTGAGCT | XM_005902845.2 | 140bp |

**S3. The gene name, sequences of PCR product, accession and product size for each gene**

^*^ LPL, lipoprotein lipase; ACACA, acetyl-CoA carboxylase; PPARγ, peroxisome proliferator-activated receptors gamma; FASN, fatty acid synthase; FABP4, adipocyte fatty acid binding protein 4; SCD, stearoyl-CoA desaturase; SREBP-1c, sterol regulatory element-binding protein-1c.
